# Clinical application of vancomycin TDM in ventilated patients with gastrointestinal cancer: a propensity-matched analysis

**DOI:** 10.1186/s12879-023-08885-7

**Published:** 2024-01-02

**Authors:** Xiaowu Zhang, Yulin Wu, Donghao Wang

**Affiliations:** Department of Intensive Care Unit, Tianjin Medical University Cancer Institute and Hospital, National Clinical Research Center for Cancer, Key Laboratory of Cancer Prevention and Therapy, Tianjin’s Clinical Research Center for Cancer, Huan-Hu-Xi Road, Ti-Yuan-Bei, He Xi District, Tianjin, 300060 China

**Keywords:** Vancomycin, Gastrointestinal cancer, Therapeutic drug monitoring

## Abstract

**Background:**

Therapeutic drug monitoring (TDM) of vancomycin is widely recommended for clinical treatment. Due to the complexity of 24-h area under the curve (AUC) guided vancomycin monitoring in clinical practice, the vancomycin trough level remains the most common and practical method. The purpose of this study was designed to investigate the differences in the safety and efficacies of vancomycin TDM based on the two different monitoring methods, and further explore the clinical application of trough-guided vancomycin monitoring in patients with gastrointestinal cancer requiring mechanical ventilation.

**Methods:**

We included a total of 78 gastrointestinal cancer patients who required mechanical ventilation due to various diseases. All patients included in this study were aged 18 years or older and were treated with intravenous vancomycin therapy for more than 2 days due to documented or suspected Gram-positive bacterial infections, and have at least one available vancomycin plasma concentration. First, we compared the safety and efficacies of vancomycin TDM based on different monitoring methods as trough-guided monitoring or AUC-guided monitoring. Then, based on whether the initial vancomycin concentration achieving the target trough concentration (less than 48 h), patients were divided into early and delayed groups, and the clinical factors were compared between them. The primary endpoints include the incidence of new-onset acute kidney injury (AKI) or renal replacement therapy (RRT), clinical success rate and 28-day all-cause mortality. Finally, the overall relationship between trough concentration and potential covariates is screened by univariate and multivariate analysis to explore potential information covariates.

**Results:**

The research revealed that patients with gastrointestinal cancer exhibited significantly lower initial vancomycin trough concentrations (median [interquartile range (IQR)]: 6.90[5.28-11.20] mg/L). And there were no statistically significant differences in the safety and efficacies of vancomycin TDM based on the two different monitoring methods for the primary endpoint. Moreover, base on trough-guided vancomycin monitoring, the early group demonstrated a notably shorter duration of mechanical ventilation compared with the delayed group (χ^2^ = 4.532; *p* < 0.05; Fig. 2E). Propensity score weighting further confirmed that the duration of mechanical ventilation (χ^2^ = 6.607; *p* < 0.05; Fig. 2F) and duration of vasoactive agent (χ^2^ = 6.106; *p* < 0.05; Fig. 2D) were significantly shorter in the early group compared with delayed group. Multivariate regression analysis revealed that Cystatin C (Cys-C) was the most important variable for vancomycin target trough achievement (odds ratio, 5.274; 95% CI, 1.780 to 15.627; *p* = 0.003).

**Conclusions:**

Trough-guided vancomycin monitoring is a simple and effective marker of TDM for ventilated patients with gastrointestinal cancer. Timely achievement of target trough concentrations for vancomycin can improve partial clinical outcomes in Gram-positive bacterial infections. Cys-C level is a potentially valuable parameter for predicting the vancomycin concentration.

## Introduction

Worldwide, gastrointestinal malignancies are the leading cause of cancer-related mortality [1]. For example, colorectal cancer (CRC) ranks third in incidence and second in mortality, while stomach cancer (GC) and esophageal cancer (EC) rank fourth and sixth in mortality, respectively [1]. However, due to inadequate nutrient intake caused by primary anorexia or gastrointestinal symptoms, systemic inflammatory syndrome and alternative substance metabolism caused by cancer, the malnutrition rate in patients with gastrointestinal cancer has significantly increased [2]. Malnutrition contributes to loss of weight and muscle, immune function decline, and ultimately increased infections, prolonged hospitalization, and increased mortality [3, 4]. Gram-positive bacterial infections are considered one of the major causes of mortality and morbidity in patients with malignant tumors. Vancomycin is a glycopeptide antibiotic widely used in clinical practice for severe infections caused by Gram-positive bacteria [5]. It inhibits bacterial growth by hindering the synthesis of cell wall in bacteria, and has strong antibiotic effect on Gram-positive bacteria included *methicillin-resistant Staphylococcus aureus* (MRSA), *Enterococcus*, or *methicillin-resistant Staphylococcus epidermidis* (MRSE) [6]. Intravenous vancomycin mainly combined with albumin and IgA, protein-bound protein is 25% to 50%, almost completely eliminated by the renal pathway. The main safety issue with vancomycin is acute kidney injury (AKI). Even mild AKI can prolong hospitalizations, increase health care costs, and increase morbidity [7]. Previous research confirmed that empirical antibacterial therapy deficiency may significantly increase infection-related morbidity and mortality of patients with malignant tumors. Still more ominously, recent evidence indicates that more than 50% of cancer patients who using vancomycin in the clinic fails to reach target concentration [8, 9]. Therapeutic drug monitoring (TDM) as an optimizing vancomycin therapy is widely recommended for avoiding secondary clinical complications because of its narrow therapeutic window, such as vancomycin toxicity due to over-dosing or resistance due to under-dosing. The 2009 vancomycin therapeutic guideline includes recommendations for TDM to control trough concentrations (C_trough_) of vancomycin. The vancomycin monitoring recommendation was to target trough levels of 10 to 20 mg/L [10]. Notwithstanding, the 2020 vancomycin therapeutic guideline for severe MRSA infections recommend monitoring the area under the curve (AUC) to the minimum inhibitory concentration (MIC) measured by a broth microdilution (BMD; AUC from 0 to 24 h [AUC_0–24_]/MIC) rather than C_trough_ [5, 11]. Meanwhile, Zhai et al. proposed that monitoring AUC24 is not suitable for patients with unstable renal function. Previous research has corroborated that cancer patient exhibit aberrant vancomycin pharmacokinetics (PKs) and pharmacodynamics (PDs) [12], however, no specific recommendations for vancomycin monitoring are recommended in these patients. Due to the potential difficulties in availability of the Bayesian method and the feasibility of using the first-order PK equation to collect 2 steady-state samples, it is recommended to use the same intensity trough concentration and AUC24 [11]. Similar recommendations were also made by the European Society of Intensive Care Medicine (ESICM) and the Anti-infectives Committee of the International Association of Therapeutic Drug Monitoring and Clinical Toxicology (IATDMCT)[13, 14]. The purpose of this study was designed to investigate the differences in the safety and efficacies of vancomycin TDM based on the two different monitoring methods, and further explore the clinical application of trough-guided vancomycin monitoring in ventilated patients with gastrointestinal cancer.

## Methods

### Study design and patient population

**Fig. 1:**
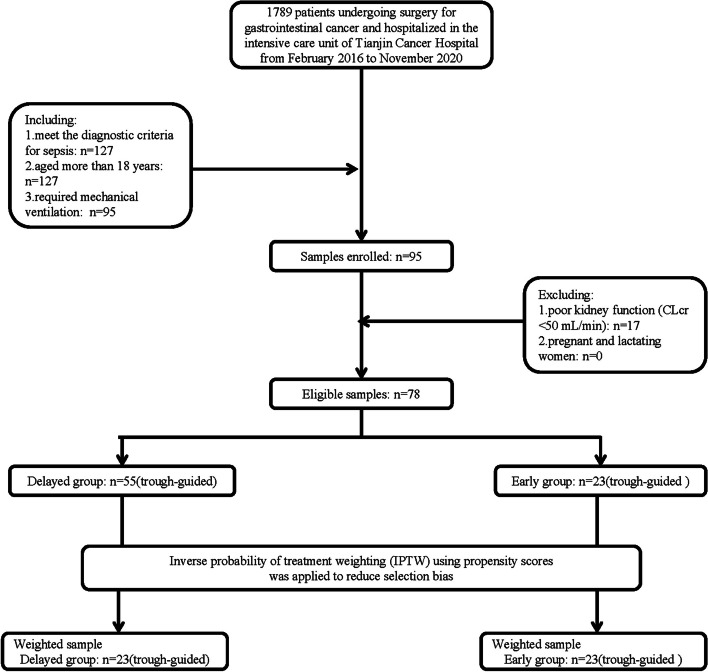
Enrollment of eligible samples for the study cohort

This study was a retrospective, propensity-matched cohort study. From February 2016 to November 2020, we retrospectively reviewed 78 patients with gastrointestinal cancer who were treated in the intensive care unit of Tianjin Cancer Hospital (Tianjin, China). The inclusion criteria as: (1) aged more than 18 years, (2) required mechanical ventilation, (3) satisfied the sepsis-III criteria and having a Sequential Organ Failure Assessment (SOFA) score of 2 or more, (4) were treated with intravenous vancomycin therapy for more than 3 days due to documented or suspected Gram-positive bacterial infections. And exclusion criteria as: (1) Human subjects with poor kidney function (CLcr < 50 mL/min), (2) received a single dose of vancomycin, (3) hemodialysis patients, (4) pregnant and lactating women. All of the enrolled patients in clinical trials received vancomycin therapy for more than five estimated terminal disposition half-lives, by which time serum vancomycin levels were supposed to have reached a steady state. Other anti-infective treatments were administered according to the patient's condition. And their data were collected from the clinical charts. This clinical trial was agreed by the Ethics Committee and approval from the Ethics Committee of Tianjin Medical University Cancer Institute and Hospital and complied with the Declaration of Helsinki. The flow diagram is shown in Fig. 1.

### Vancomycin administration and therapeutic drug monitoring (TDM)

The vancomycin dosage was administered over a 1-h period. The steady state vancomycin trough concentration was measured prior to subsequent treatment to adjust dose and dose intervals. The target serum vancomycin trough concentrations ranged from 10-15 or 15-20 mg/L [5]. Serum vancomycin concentrations were measured by fluorescence polarization immunoassay (FPIA) method using a Cobas 6000 c501 analyzer (Roche Diagnostics, China). The AUC was calculated by the classic pharmacokinetic software Vancomycin Calculator (https://clincalc.com/Vancomycin), which supports estimating AUC by a Bayesian approach based on trough data only. The laboratory staff records information about each specimen, including gender, age, body weight, serum creatinine concentration, daily dosage, dose interval, infusion time, sampling time since infusion end and measure concentration for pharmacokinetic analysis.

### Data collection, study definitions

Demographic data obtained included age, gender, admission diagnosis, acute physiology and chronic health assessment II score (Apache II score) at admission to the ICU. For the treatment program, daily doses, interval time, concomitant antibiotics, and the occurrence of acute kidney injury (AKI) and renal replacement therapy (RRT) were recorded. New-onset acute kidney injury was defined according to the KDIGO (Kidney Disease: Improving Global Outcomes) stage II criteria after at least 24 h and within 7 days of vancomycin administration initiation. The duration of the antibiotic and the vasoactive agent, the duration of mechanical ventilation, and the 28-day all-cause mortality were also recorded. Finally, based on whether the initial vancomycin concentration achieving the target trough concentration (less than 48 h), the patients were divided into early and delayed groups. Inverse probability of treatment weighting (IPTW) was used to measure participants based on the estimated exposure probability (the propensity score) for a given confounding factor to balance the observed confounding factors between two groups [15]. The primary endpoints include the incidence of new-onset AKI or RRT, clinical success rate and 28-day all-cause mortality between two groups (early group vs. delayed group). Secondary endpoints include the duration of mechanical ventilation, the duration of antibiotics and the duration vasoactive agent. The overall relationship between trough concentrations and potential covariates was screened by Univariate and multivariate analysis to explore potential information covariates.

### Statistical analysis

Values for categorical variables are given as count (percentage), for continuous variables, as mean ± standard deviation or as median (interquartile range). Statistical differences between continuous results were tested using Student’s t test or the Mann–Whitney U test. The chi-square test or Fisher’s exact test was used for categorical results. Survival was estimated by the Kaplan-Meier method and compared using the log-rank test. The propensity score (PS) was calculated using a multivariable logistic regression model with the two groups. Inverse probability of treatment weight (IPTW) was then calculated using PS. Relative risk was estimated using odds ratios (ORs) with corresponding 95% confidence intervals. Univariate and multivariate analysis is used for covariates associated with target trough achievement. All statistical analyses were performed using the SPSS statistical package (version 24.0, SPSS Inc., Chicago), *p* < 0.05 was considered statistically significant.

## Results

**Fig. 2:**
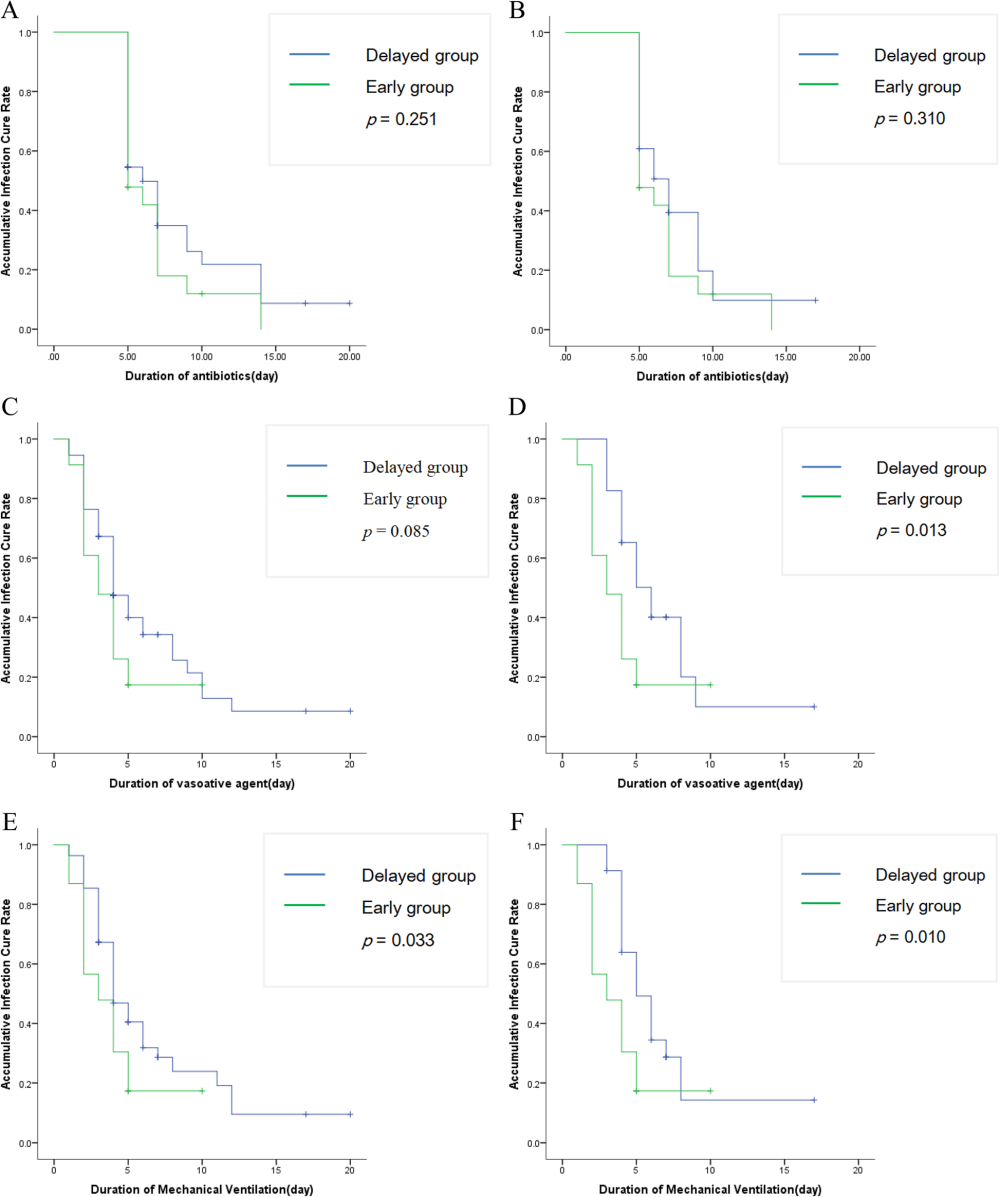
Kaplan-Meier curves for (**A**) Duration of Antibiotics between the two groups before weighting; (**B**) Duration of Antibiotics after weighting; (**C**) Duration of Vasoactive Agent between the two groups before weighting; (**D**) Duration of Vasoactive Agent after weighting; (**E**) Duration of Mechanical Ventilation between the two groups before weighting; (**F**) Duration of Mechanical Ventilation after weighting

A total of 1789 patients were admitted to the ICUs during the study period. And 78 patients were enrolled, all of whom received recommended standard vancomycin dosage adjustment. Clinical characteristics, drug dosage and vancomycin trough concentration were summarized in Tables 1 and 2. Our research revealed that the patients with gastrointestinal cancer have a significantly lower initial vancomycin trough concentration (median [IQR]: 6.90 [5.28-11.20] mg/L) than the recommended standard vancomycin trough concentrations (10-15 or 15-20 mg/L) [5]. We compared the safety and efficacies of vancomycin TDM based on different monitoring methods as trough-guided monitoring or AUC-guided monitoring, and there were no statistically significant difference between the 2 groups for the vancomycin-induced AKI or RRT (*p* = 0.960), clinical success rate(*p* = 1.000) and 28-day all-cause mortality(*p* = 0.489) (Table 3). It should be noted that the Cys-C was statistically significantly higher in trough-guided TDM group (*p* = 0.029). Then, we divided patients into early group and delayed group based on whether the initial trough concentration has achievedd the target concentration. It is noteworthy that there was a statistical difference in Cys-C between the early and late groups(*p* < 0.001). Similar results were also found after IPTW method(*p* < 0.019). The primary endpoint were similar between two groups in Table 4 (e. g., clinical success rate, 28-day all-cause mortality, the incidence of new-onset AKI or RRT), but the duration of mechanical ventilation in Early group was considerably shorter compared with Delayed group (χ^2^ = 4.532; *p* < 0.05; Fig. 2E). However, there was no difference between the two groups in duration of antibiotics (χ^2^ = 1.318; *p* = 0.251; Fig. 2A, B) and of duration vasoactive agent (χ^2^ = 2.941; *p* = 0.086; Fig. 2C). After IPTW method, the duration of mechanical ventilation in Early group was considerably shorter compared with Delayed group (χ^2^ = 4.532; *p* < 0.05; Fig. 2E). Compared with Delayed group, propensity score weighting (IPTW) further confirmed that the duration of mechanical ventilation (χ^2^ = 6.607; *p* < 0.05; Fig. 2F) and vasoactive agent (χ^2^ = 6.106; *p* < 0.05; Fig. 2D) in Early group were considerably decreased. The overall relationship between trough concentrations and potential covariates was screened by univariate and multivariate analysis to explore potential information covariates. Univariate analysis showed a strong correlation between vancomycin trough concentration and age (*p* = 0.039), body weight (*p* <  = 0.043), serum creatinine (*p* = 0.032) and serum Cys-C (*p* = 0.001) (Table 5). Multivariate regression analysis revealed that the Cys-C was the most important variable for vancomycin target trough achievement (odds ratio, 5.274; 95% CI, 1.780 to 15.627; *p* = 0.003) (Table 5). For Delay group, all the patients made vancomycin dosage adjustment and repeated TDM daily until achieved the target therapeutic concentration.

**Table 1:** Characteristics of patients who determined vancomycin therapeutic drug monitoring program (*n* = 78)

Characteristics	
Sex (male/female)	17/61
Age (years)	65 [57–63]
Body weight (kg)	79 [71.5–86]
Apache II score	19 [18-22]
SOFA score	13 [12-15]
Albumin (g/L)	28.20 [24.85–32.30]
Serum creatinine (μmol/L)	56.0 [46.0–84.5]
Cys-C (mg/L)	1.105 [0.770–1.418]
**Cancer type, n(%)**	
Hepatocellular carcinoma	16 (20.5)
Pancreatic cancer	6 (7.7)
Colorectal cancer	22 (28.2)
Gastric cancer	32 (41.0)
Gallbladder cancer	2 (2.6)
**Tumor stage, n(%)**	
I	0 (0)
II	7 (9.0)
III	56 (71.8)
IV	15 (19.2)
**Suspected source of infection, n(%)**	
Pulmonary	39 (50.0)
Intra-abdominal	28 (35.9)
Surgical incision	3 (3.8)
Other/unknown	8 (10.3)
**Identified pathogens, n(%)**	
*methicillin-resistant Staphylococcus aureus*	11 (14.1)
*methicillin-resistant Staphylococcus epidermidis*	16 (20.5)
*methicillin-sensitive Staphylococcus aureus*	17 (21.8)
*Enterococcus*	29 (37.2)
Other Gram-positive *coccus*	5 (6.4)
**Concomitant antibiotics**^**a**^**, n(%)**	
Beta-lactam	31 (39.7)
Carbapenem	47 (60.3)
Antifungal	8 (10.3)
None	3 (3.8)

*Abbreviations*: *APACHE II* acute physiology and chronic health evaluation II, *SOFA* Sequential Organ Failure Assessment, *Cys-C* Cystatin C

*Note*: ^a^Beta-lactam drugs included Piperacillin-Tazobactam and Cefoperazone-Sulbactam; Carbapenem drugs included Meropenem and Imipenem-Cilastatin; Antifungal drugs included Fluconazole, Voriconazole and Caspofungin

Values for categorical variables are given as count (percentage); values for continuous variables as median [interquartile range]

**Table 2:** Vancomycin dosing, distribution of C_trough_ and AUC, Primary and secondary endpoints (*n* = 78)

Characteristic	
**Initial dose (daily), n (%)**	
1000–2000 mg	4 (5.1)
2001–3000 mg	50 (64.1)
> 3000 mg	24 (30.8)
Average daily dosage (mg/kg, q12h)	15.18 ± 3.29
	15.38 [14.29–19.27]
Average trough concentration (mg/L)	8.26 ± 5.01
	6.90 [5.28–11.20]
C_trough_, n (%)	
< 10 mg/L	54 (69.2)
10–15 mg/L	15 (19.2)
15–20 mg/L	8 (10.3)
> 20 mg/L	1 (1.3)
**AUC, n (%)**	
< 400	52 (66.7)
400–600	24 (30.7)
> 600	2 (2.6)
**Clinical Outcomes**	
***Primary endpoints***	
Clinical success rate, n (%)	58 (74.4)
28-day all-cause mortality, n (%)	4 (5.1)
Composite outcome of new-onset AKI or RRT, n (%)	7 (9.0)
***Secondary endpoints***	
Duration of vasoactive agent (day)	4 [2-5]
Duration of ventilation (day)	4 [3-5]
Duration of antibiotics (day)	5 [5-7]

*Abbreviations*: *C*_trough_ trough concentration, *AKI* acute kidney injury, *RRT* renal replacement therapy

*Note*: Values for categorical variables are given as count (percentage); values for continuous variables, as mean ± standard deviation or median [interquartile range]

**Table 3:** Comparison of pharmacokinetic parameters and patient demographics between C_trough_ and AUC

Characteristics	C_trough_ = 10-20 mg/L	AUC = 400–600	χ^2^	*p* value
	(*n* = 23)	(*n* = 24)		
Age, years				
Mean ± S.D	68.78 ± 10.45	71.75 ± 8.497		0.290^a^
Median [range]	68 [61–76]	73 [64–78]		
Sex, n(%)				1.000^c^
Male	19 (82.6)	20 (83.3)		
Female	4 (17.4)	4 (16.4)		
Body weight, kg				
Mean ± S.D	81.61 + 9.30	84.58 + 8.17		0.250^a^
Median [range]	84 [75–88]	86 [77–91]		
Apache II score on ICU				
Mean ± S.D	19.65 + 3.60	18.75 + 2.85		0.345^a^
Median [range]	20 [17–22]	18 [17–20]		
SOFA score				
Mean ± S.D	13.17 + 1.88	12.88 + 2.19		0.619^a^
Median [range]	13 [12–14]	13 [11–14]		
Albumin (g/L)				
Mean ± S.D	28.10 + 6.53	27.58 + 4.81		0.754^b^
Median [range]	29.40 [23.70–33.50]	28.30 [23.80–32.30]		
Serum creatinine (μmol/L)				
Mean ± S.D	79.00 + 26.24	70.92 + 32.14		0.351^b^
Median [range]	76.00 [63.00–89.00]	59.50 [49.00–92.00]		
Cys-C (mg/L)				
Mean ± S.D	1.67 + 0.56	1.32 + 0.51		0.029*^a^
Median [range]	1.60 [1.25–1.90]	1.21 [0.87–1.84]		
**Clinical Outcomes**				
Clinical success rate, n (%)	19 (82.6)	19 (79.2)	0.000	1.000^c^
28-day all-cause mortality, n (%)	0	2 (8.3)		0.489^d^
Composite outcome of new-onset AKI or RRT, n (%)	3 (13.0)	2 (8.3)	0.003	0.960^c^

*Abbreviations*: *C*_trough_ trough concentration, *APACHE II* acute physiology and chronic health evaluation II, *SOFA* Sequential Organ Failure Assessment, *Cys-C* Cystatin C

*Note*: ^a^Student’s t test

^b^Mann–Whitney U test

^c^Pearson χ^2^ test

^d^Fisher’s Exact Test

Values for categorical variables are given as count (percentage); values for continuous variables, as mean ± standard deviation or median [interquartile range]

^*^p < 0.05

**Table 4:** Baseline data and primary endpoints of patients before and after propensity score weighting (*n* = 78)

	Entire cohort				Propensity score weighting			
Characteristics	Delayed group	Early group	χ^2^	*p*	Delayed group	Early group	χ^2^	*p*
	(*n* = 55)	(*n* = 23)			(*n* = 23)	(*n* = 23)		
Age, years								
Mean ± S.D	62.65 ± 11.87	68.78 ± 10.45		0.035*	65.22 ± 10.76	68.78 ± 10.45		0.260
Median [range]	65 [56–70]	68 [61–76]			67 [60–74]	68 [61–76]		
Sex, n(%)				0.542				0.381
Male	42 (76.4%)	19 (82.6%)			21 (91.3%)	19 (82.6%)		
Female	13 (23.6%)	4 (17.4)			2 (8.7%)	4 (17.4)		
Body weight, kg								
Mean ± S.D	75.53 ± 12.38	81.61 ± 9.302		0.038*	78.52 ± 11.63	81.61 ± 9.30		0.326
Median [range]	77 [69–83]	84 [75–88]			80 [72–87]	84 [75–88]		
Apache II score on ICU								
Mean ± S.D	19.51 ± 2.73	19.65 ± 3.60		0.849	20.00 ± 2.73	19.65 ± 3.60		0.714
Median [range]	19 [18–22]	20 [17–22]			20 [17–22]	20 [17–22]		
SOFA score								
Mean ± S.D	13.15 ± 2.41	13.17 ± 1.87		0.974	13.70 ± 2.55	13.17 ± 1.87		0.307
Median [range]	13 [11–15]	13 [12–14]			13 [12–15]	13 [12–14]		
Albumin (g/L)								
Mean ± S.D	27.86 ± 5.50	28.10 ± 6.53		0.867	27.24 ± 3.70	28.10 ± 6.53		0.587
Median [range]	28.0 [25.7–32.3]	29.4 [23.7–33.5]			27.7 [25.7–29.4]	29.4 [23.7–33.5]		
Serum creatinine (μmol/L)								
Mean ± S.D	62.73 ± 29.81	79.00 ± 26.24		0.026*	81.09 ± 32.46	79.00 ± 26.24		0.812
Median [range]	50 [42–77]	76 [63–89]			74 [53–105]	76 [63–89]		
Cys-C (mg/L)								
Mean ± S.D	1.11 ± 0.64	1.66 ± 0.56		0.001*	1.30 ± 0.48	1.67 ± 0.56		0.019*
Median [range]	0.90 [0.74–1.24]	1.60 [1.25–1.90]			1.21 [0.98–1.56]	1.60 [1.25–1.90]		
**Primary endpoints**								
Clinical success rate, n(%)	39 (70.9)	19 (82.6)	1.164	0.281	16 (69.6)	19 (82.6)	1.075	0.300
28-day all-cause mortality,n(%)	4	0	0.585	0.444	3 (13.0)	1 (4.3)	0.274	0.601
Composite outcome of new-onset AKI or RRT, n(%)	4 (7.3)	3 (13.0)	0.143	0.705	4 (17.4)	3 (13.0)	0.000	1.000

*Abbreviations*: *APACHE II* acute physiology and chronic health evaluation II, *SOFA* Sequential Organ Failure Assessment, *AKI* acute kidney injury, *RRT* renal replacement therapy

*Note*: Values for categorical variables are given as count (percentage); values for continuous variables, as mean ± standard deviation or median [interquartile range]

^*^p < 0.05

**Table 5:** Univariate and multivariate analysis of covariates associated with target trough achievement

Variants	Univariate analysis			Multivariate analysis		
	OR	95% CI	*p*	OR	95% CI	*p*
Sex	1.470	0.423–5.105	0.544			
Age (years)	1.057	1.003–1.114	0.039*	1.152	0.997–1.331	0.054
Body weight (kg)	1.057	1.002–1.116	0.043*	0.929	0.804–1.073	0.315
Apache II score	1.016	0.863–1.198	0.846			
SOFA score	1.006	0.809–1.250	0.959			
Albumin (g/L)	1.007	0.925–1.097	0.864			
Serum creatinine (μmol/L)	1.018	1.002–1.035	0.032*	1.018	0.993–1.045	0.158
Cys-C (mg/L)	5.249	1.972–13.972	0.001*	5.274	1.780–15.627	0.003*

*Abbreviations*: *APACHE II* acute physiology and chronic health evaluation II, *SOFA* Sequential Organ Failure Assessment, *Cys-C* Cystatin C

^*^p < 0.05

## Discussion

Among these 78 patients with gastrointestinal cancer, there was no statistically significant difference between trough-guided and AUC-guided TDM for the primary endpoint, including Clinical success rate, 28-day all-cause mortality and new-onset AKI or RRT. And the results indicated that vancomycin trough concentration would be a simple and effective TDM marker for these patients. This would be helpful, especially for source-limited settings. The results of this study once again confirm that cancer patients have low levels of vancomycin [12], and small number of these 78 patients achieved the target trough concentration. Several explanations have been offered to explain these findings. One reason include that physiological processes are altered in patients with cancer and may alter VCM PKs [16–19]. Alqahtani S et al. confirmed that patients with cancer showed a significantly higher vancomycin clearance than noncancer patients, whereas the volume of distribution (V) was found to be similar in both groups [12]. Curth et al. showed that patients with cancer could require an ~ 50% higher dose than the normal. This explanation is backed by studies that direct activation of renal organic anion/cation transporters (OCT1/2, OATs) by cytokines such as TNF-α leads to an increase in renal clearance, which is confirmed by in vivo experiments [20, 21]. Recently, some studies have proposed augmented renal clearance (ARC) to describe the enhancement of renal elimination of circulating solutes observed in critically ill patients [22].

Our research also reveals that the time to achieve the target concentration of vancomycin is associated with partial clinical outcomes, including shorter duration of mechanical ventilation (χ^2^ = 6.607; *p* < 0.05), and shorter duration of vasoactive agent (χ^2^ = 6.106; *p* < 0.05), which means earlier hemodynamic stability. Previous research confirmed that higher loading dose of vancomycin is necessary to achieve ideal therapeutic level of vancomycin. Unfortunately, this approach has the consequence of higher rates of AKI, which increases mortality. And the only modifiable risk factor for AKI was vancomycin trough level of > 30 mg/L. Fortunately, TDM affords clinicians the opportunity to assess whether the dose they are providing is achieving the expected serum concentration, which may minimize the risk of nephrotoxicity and to ensure successful therapeutic outcomes.

In this research, we also observed a strong correlation between vancomycin trough concentrations and the Cys-C, which is a non-glycosylated, low molecular weight basic protein composed of 120 amino acids [23, 24]. Previous studies mainly calculated eGFR via creatinine-based equations. However, creatinine is affected by factors such as age, muscle mass and diet, and is thus a less sensitive marker of renal function compared to Cys-C [25]. As an alternative biomarker for glomerular filtration rate (GFR), Cys-C has attracted great research interest, and many studies have been conducted in recent years to evaluate Cys-C [26–28]. Cys-C is a low-molecular-weight protein that is produced stably in the body and has been proposed as an alternative endogenous marker of glomerular filtration. It is the product of a housekeeping gene expressed in all nucleated cells, thus exhibiting a stable production rate irrespective of muscle mass, gender, and body weight, even in the presence of acute inflammatory responses [29]. Thus, Cys-C has many characteristics of an ideal endogenous GFR marker. Recent studies have shown that Cys-C as a GFR marker may be superior to Serum creatinine (SCr) [30]. Our studies also found that in gastrointestinal cancer patients, the correlation between vancomycin concentration and serum Cys-C (odds ratio, 5.274; 95% CI, 1.780 to 15.627; *p* = 0.003) is better than that between serum creatinine concentration (odds ratio, 1.018; 95% CI, 0.993 to 1.045; *p* = 0.158) and vancomycin concentration. In this study, we observed that the level of Cys-C in the early group was significantly higher than that in the late group. Considering that Cys-C is a better marker of GFR than SCr. This increase may indicate potential renal insufficiency in the early group, which is also a potential reason for the early group to achieve the target trough level preferentially. This parameter allows doctors or pharmacists to predict vancomycin dose requirements in a short period of time and optimize the adequacy of antimicrobial drugs.

Despite these advantages, there are several potential limitations in research. Firstly, due to the fact that it is a single-center retrospective study, the sample size was relatively small which leads to lower statistical power to detect significant associations. However, given that this is an exploratory study and some meaningful results have been obtained, we are designing a clinical trial with a larger sample size and longer follow-up time based on these promising results. Meanwhile, the retrospective design of the study prevents the investigation of different clinical and microbiological outcomes, nephrotoxicity, and cost. Secondly, although estimation of AUC based on trough concentration only was recommended by guidelines, the trough-only estimates would be less accurate compared to two-sample strategy and intensive sampling, and the results may vary when implying different PPK models. Thirdly, this study was at a single center, which has the advantage of eliminating potentially confounding site specific factors, but a potential disadvantage for generalization of the results to other facilities. Finally, inappropriately timed vancomycin trough concentration determination is a general challenge for therapeutic drug monitoring and is present regardless of the vancomycin administration algorithm used.

## Conclusion

Trough-guided vancomycin monitoring is a simple and effective marker of TDM for ventilated patients with gastrointestinal cancer. Since initial trough concentrations in these populations are significantly reduced, clinicians should pay particular attention to changes in vancomycin pharmacokinetic. Minimizing the time to initial vancomycin target trough can improve partial clinical outcomes in gram positive infections, and special dosage regimens are needed to achieve this clinical purpose. The Cys-C level is a potentially valuable parameter for predicting vancomycin concentration, and Cys-C inclusive dosing models are warranted.

## Data Availability

The datasets used and/or analyzed during the current study are available from the corresponding author on reasonable request.
